# WHO CAN USE YOUR CELLS OR TISSUES FOR SCIENTIFIC RESEARCH?

**DOI:** 10.3389/frym.2024.1233752

**Published:** 2024-01-31

**Authors:** Jessica L. Beers, Raeanne M. Geffert, Bethany D. Latham, Klarissa D. Jackson

**Affiliations:** Division of Pharmacotherapy and Experimental Therapeutics, University of North Carolina at Chapel Hill Eshelman School of Pharmacy, Chapel Hill, NC, United States

## Abstract

Informed consent is the process of obtaining permission from human participants to use their cells and tissues or otherwise include them in research studies. With informed consent, scientists can use human cells or tissues in experiments to learn more about the human body and to test new medicines. This article describes how these tissues are obtained, and the ethical concerns regarding the use of human tissues in research. The story of Henrietta Lacks and her immortal HeLa cell line is discussed, to demonstrate the importance of informed consent and to showcase Henrietta’s valuable contributions to research and modern medicine.

## WHAT IS HUMAN TISSUE RESEARCH?

Scientists can study cells or tissues from the human body in a lab to learn more about how the body works. These tissues can be blood, parts of organs, or cells. Tissues can be collected from living or dead patients, depending on the organ that is being studied [1].

Tissue research requires **informed consent** from the patient—basically the patient needs to understand what will be studied and say it is OK to use their tissues. Informed consent for tissue research must be obtained without **coercion**: coercion is a way of persuasion, usually with a threat or force [2]. Without informed consent from the patient, tissues cannot be used in any type of research. Tissue research has provided many advances in human medicine [2]. It is important to understand where tissues come from, how patients are involved, and the scientific advancements that have been made from human tissue research.

## WHY DO WE USE HUMAN TISSUES IN SCIENTIFIC RESEARCH?

An **in vitro** experiment is one that is conducted with cells or tissues from the body, but the experiment is done *outside* of the body, such as in a test tube or culture dish. Scientists conduct *in vitro* research because research with cells and tissues allows them to perform experiments they could not perform on humans [1]. These experiments can use test drugs or toxic chemicals that might harm living participants, but because tissues are removed from the body, researchers can use them to learn more about how drugs or chemicals might affect a person.

## HOW ARE HUMAN TISSUES COLLECTED FOR RESEARCH?

Scientists can collect donated tissues that are left over from medical procedures such as surgeries, or from people who agree to donate their tissues for research after their death. These samples would otherwise be discarded if they were not used for research. Once the sample is collected, it is quickly transported to a lab and preserved at very cold temperatures, or experiments are performed right away with fresh samples. It is important for researchers to preserve donated tissues quickly since cells and tissues can break down outside the human body, which may alter the results of experiments. Research companies may also collect and store donated tissues that scientists can then purchase for their own research.

## HOW DO RESEARCHERS PROTECT THE RIGHTS OF HUMAN TISSUE DONORS?

There are several important **ethical** concerns in using human cells and tissues for research. These include obtaining permission from donors and protecting their privacy. Fortunately, there are laws and guidelines in place to ensure that the rights of donors are protected.

In the United States, the Health Insurance Portability and Accountability Act (HIPAA) is a law that protects the privacy of donors by requiring samples to be **de-identified** after they are donated [3]. This means that samples are labeled so that researchers do not know the identity or personal health information of the donors [3]. Instead, researchers may only know basic information such as the age, sex, and race of the donor [3].

**Figure 1:**
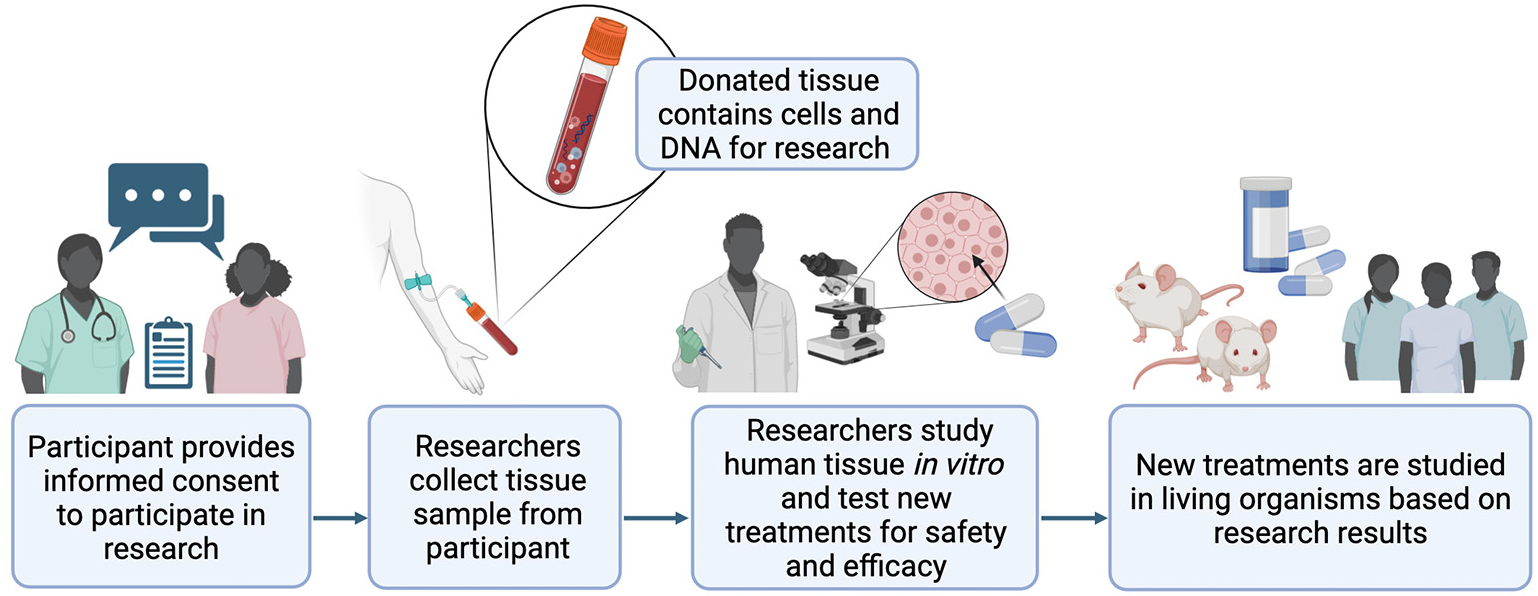
Research on cells and tissues outside of a living organism, called *in vitro* research, allows scientists to perform experiments they could not perform on humans, like testing new medicines for safety. Doctors and scientists must obtain informed consent from donors before they take any tissues for research use. This means that the donors must be informed about how their samples will be used, and they have the right to say no if they do not want to participate. Created with BioRender.com.

In addition, samples are collected only with the permission of an **Institutional Review Board** (IRB) at the location where the samples are collected [4]. The IRB makes sure donors are adequately informed about how and why their samples will be collected. The IRB also ensures that donors give informed consent to researchers before their tissues are collected. During the informed consent process, participants speak directly with research study staff, and they are informed of any potential risks or benefits associated with the research. Participants are encouraged to ask questions about their role in the research before choosing whether to donate their tissues. Participation in the research is completely the participant’s choice. Throughout the study, participants may continue to ask questions and can choose to leave the study at any time without a penalty. Regardless of whether a patient decides to participate in a study or donate their tissues for research, the quality of their healthcare will not be affected. Figure 1 shows the process of how human tissues are collected and used for research.

Over the years, there has been disagreement about how humans and their cells can be used for research. In 1964, the World Medical Association published the Helsinki Declaration, which made rules to protect humans in research all over the world [5]. Some people have still tried to take advantage of others to get research results. One example is the Tuskegee study, a 40-year-long study in which hundreds of patients with a disease called syphilis were left untreated even though treatment was available [5]. Once news of this study reached the public, the United States released the Belmont Report in 1976. This made the rules for research in the United States today and has three main principles [4, 5]. The first principle is respect, which means that all researchers must take the person’s feelings and choices into consideration when they interact with the person or their tissues. The second is beneficence, which means that the person must benefit from the study in a way that improves their health or life. The last principle is justice, which means that each person must be treated fairly, no matter who they are or what they look like. These rules must be followed by every researcher in the United States.

## HENRIETTA LACKS AND HER IMMORTAL CELLS

In 1951, Henrietta Lacks was a young African American woman who lived in Baltimore, Maryland, and who had cervical cancer. She was treated at the Johns Hopkins Hospital in Baltimore. During her treatment, cancer cells were taken from her without her informed consent or knowledge. The doctors and scientists who used her cells never asked Henrietta if she wanted to donate cells. These cells (known as HeLa cells, for Henrietta’s name) were transformed in a lab into an **immortal cell line** [6]. An immortal cell line can multiply and continue to live outside the human body for long periods of time. This was an incredible discovery and very important for the future of human tissue research. Today, immortal cell lines are relatively easy to use, low cost, and allow researchers an unlimited supply of cells when grown correctly.

It is important to note that all human cells contain identifiable genetic information, and these cells will carry this information as long as they are kept alive by cell cultures. Henrietta’s immortal cell line therefore contains information that is specific to her, and that information is similar to that of her relatives. Today, researchers must get permission to collect genetic information and must keep this information private. As an African American woman, Henrietta was from a historically excluded group, especially in healthcare. She was not given credit for her cells until long after many scientific discoveries were made using them, and she and her family never received compensation for the unethical use of her cells [7]. She passed away from cancer without recognition. Her family discovered her contribution many years later, and they have been working to help celebrate her legacy in science and medicine. This is now a widely recognized example of injustice in science, and it is extremely important for scientists to remember, as they continue to use HeLa cells in research today.

## THE LEGACY OF HENRIETTA LACKS

Due to Henrietta Lacks’ contribution to modern medicine, millions of lives have been saved. Many vaccines, such as those for polio and COVID-19, were created using her cells [6]. Treatments for deadly diseases like HIV/AIDS, sickle cell anemia, and many types of cancer were discovered by scientists using HeLa cells [7]. This is why Henrietta Lacks is referred to as the mother of modern medicine. However, as we mentioned, she has not always been given credit for her contributions to science. Although it was legal at the time, it was unethical for her doctor to use her cells in this way without her informed consent [7]. Henrietta’s story is important because scientists of the future must know the wrongs committed by previous generations. This knowledge helps us learn from past mistakes and contributes to ethical scientific research that exhibits **racial equity**. Today, the Belmont Report protects humans in research [4, 5]. Informed consent ensures respect for people. Justice ensures that the benefits and risks of research are distributed equally, without prejudice toward members of a particular gender or race. It is because of Henrietta Lacks that people of all ethnicities can have access to life-saving treatments and benefit from new scientific discoveries.
